# Cervicovaginal DNA Virome Alterations Are Associated with Genital Inflammation and Microbiota Composition

**DOI:** 10.1128/msystems.00064-22

**Published:** 2022-03-28

**Authors:** Emily A. Kaelin, Peter T. Skidmore, Paweł Łaniewski, LaRinda A. Holland, Dana M. Chase, Melissa M. Herbst-Kralovetz, Efrem S. Lim

**Affiliations:** a School of Life Sciences, Arizona State University, Tempe, Arizona, USA; b Center for Fundamental and Applied Microbiomics, The Biodesign Institute, Arizona State University, Tempe, Arizona, USA; c Department of Basic Medical Sciences, College of Medicine—Phoenix, University of Arizona, Phoenix, Arizona, USA; d Department of Obstetrics and Gynecology, College of Medicine-Phoenix, University of Arizona, Phoenix, Arizona, USA; e UA Cancer Center, University of Arizona, Tucson, Arizona, USA; Mayo Clinic

**Keywords:** cervicovaginal virome, cervical cancer, human papillomavirus, HPV, *Lactobacillus* species, genital inflammation, vaginal microbiome, anellovirus, transkingdom interactions

## Abstract

While the link between the cervicovaginal bacterial microbiome, human papillomavirus (HPV) infection, and cervical cancer is recognized (P. Łaniewski, D. Barnes, A. Goulder, H. Cui, et al., Sci. Rep. 8:7593, 2018, http://dx.doi.org/10.1038/s41598-018-25879-7; A. Mitra, D. A. MacIntyre, Y. S. Lee, A. Smith, et al., Sci. Rep. 5:16865, 2015, http://dx.doi.org/10.1038/srep16865; A. Mitra, D. A. MacIntyre, J. R. Marchesi, Y. S. Lee, et al., Microbiome 4:58, 2016, http://dx.doi.org/10.1186/s40168-016-0203-0; J. Norenhag, J. Du, M. Olovsson, H. Verstraelen, et al., BJOG, 127:171–180, 2020, http://dx.doi.org/10.1111/1471-0528.15854; E. O. Dareng, B. Ma, A. O. Famooto, S. N. Adebamowo, et al., Epidemiol. Infect. 144:123–137, 2016, http://dx.doi.org/10.1017/S0950268815000965; A. Audirac-Chalifour, K. Torres-Poveda, M. Bahena-Roman, J. Tellez-Sosa et al., PLoS One 11:e0153274, 2016, http://dx.doi.org/10.1371/journal.pone.0153274; M. Di Paola, C. Sani, A. M. Clemente, A. Iossa, et al., Sci. Rep. 7:10200, 2017, http://dx.doi.org/10.1038/s41598-017-09842-6), the role of the cervicovaginal virome remains poorly understood. In this pilot study, we conducted metagenomic next-generation sequencing of cervicovaginal lavage specimens to investigate the relationship between the cervicovaginal DNA virome, bacterial microbiome, genital inflammation, and HPV infection. Specific virome alterations were associated with features of the local microenvironment related to HPV persistence and progression to cervical cancer. Cervicovaginal viromes clustered distinctly by genital inflammation state. Genital inflammation was associated with decreased virome richness and alpha diversity and an increased abundance of *Anelloviridae* species from the genus *Alphatorquevirus*. *Lactobacillus* bacteriophages were closely associated with increased *Lactobacillus* abundance, consistent with phage-host relationships. Interestingly, bacteria-bacteriophage transkingdom interactions were linked to genital inflammation and showed specific interactions with bacterial vaginosis-associated bacteria, including *Gardnerella, Prevotella,* and *Sneathia*. Taken together, our results reveal prominent virome interactions with features of the cervicovaginal microenvironment that are associated with HPV and cervical cancer. These findings expand our understanding of the cervicovaginal host-microbiome interactions in women’s health.

**IMPORTANCE** HPV infection is an established risk factor for cervical cancer. However, more broadly, the role of the cervicovaginal virome in cervical cancer progression is not well understood. Here, we identified cervicovaginal DNA virome alterations associated with local microenvironment factors (vaginal microbiota and genital inflammation) that influence HPV persistence and progression to cervical cancer. These findings indicate that the cervicovaginal virome plays an important role in women’s health.

## INTRODUCTION

Cervical cancer is one of the most frequently diagnosed cancers among women, with more than 600,000 cases and 340,000 deaths reported worldwide in 2020 (1). Human papillomavirus (HPV) infection and immunodeficiency are well-established risk factors for cervical cancer. Other risk factors include smoking and oral contraceptive use (2, 3). Interactions between human immunodeficiency virus (HIV) and HPV are also significant, with HIV infection increasing the risk of HPV infection and persistence (4). Different HPV genotypes present various levels of risk for progression to cancer. Many high-risk genotypes belong to the genus *Alphapapillomavirus*. Other low-risk genotypes, while less studied, have been found in a variety of sites, such as nonmelanoma skin cancers and healthy skin (5). The microbiome has been shown to play an important role in cancer progression, as dysbiosis has been shown to increase HPV persistence and HPV-mediated progression to cervical cancer (6, 7). It is unclear why women respond differently to HPV infection, as there are a disproportionate number of HPV infections relative to cervical cancer cases and most infections are cleared (8). Inflammation may play a role, as it is closely tied to cancer development and progression (9). Increased levels of proinflammatory cytokines have been observed in patients with invasive cervical carcinoma, indicating an inflammatory microenvironment (10). Host-microbiome interactions may also play a key role in explaining why some women clear HPV infection while others develop a persistent HPV infection (11).

Given the importance of the microbiome in human health and disease, recent studies have focused on understanding the relationship between the vaginal microbiome and cervical cancer. The healthy vaginal bacterial microbiome is dominated by *Lactobacillus* species (12, 13). Bacterial vaginosis, characterized by low *Lactobacillus* abundance and overrepresentation of anaerobes, including *Gardnerella*, *Prevotella*, *Atopobium*, *Sneathia*, *Megasphaera*, and others, has been associated with HPV infection and cervical intraepithelial neoplasia (14–18). Previous studies have also associated low relative abundance of *Lactobacillus* species with high-risk HPV infection and invasive cervical carcinoma (10, 19). Genital inflammation and elevated vaginal pH have also been associated with cervical cancer (10).

The virome plays an important but understudied role in health and disease, as it can interact with both the host and the bacterial microbiome (20, 21). While the vaginal bacterial microbiome has been well studied, less is known about the cervicovaginal virome, especially in the context of HPV infection. Virome alterations have been linked to various clinical outcomes, such as preterm birth and bacterial vaginosis (BV) (22). Other studies have shown associations between the virome and inflammatory bowel disease and simian immunodeficiency virus infection (23, 24). Bacteriophages are a significant component of the virome as they can have a direct effect on bacterial microbiome composition (25). A recent study investigated the vaginal virome in women with BV and found a strong association between BV status and virome composition, with significant interactions between bacteriophages and the bacterial microbiome (26). These findings highlight the importance of the virome in human health and disease, particularly in women’s health.

While recent studies have greatly expanded our knowledge of the role of the vaginal microbiome in health and disease, our understanding of the vaginal virome in the context of cervical cancer is very limited. In this study, we investigated the relationship between the cervicovaginal DNA virome and other features of local microenvironment, cervicovaginal microbiota, and genital inflammation, all factors that influence HPV persistence and progression to cervical cancer. We performed viral metagenomic sequencing on physician-collected cervicovaginal lavage (CVL) samples from women in the Phoenix, AZ, metropolitan area. We found that genital inflammation and low *Lactobacillus* species abundance were associated with changes in virome alpha diversity and bacteriophage abundance. We also identified *Anelloviridae* species as being associated with the presence of genital inflammation. These data provide an opportunity to characterize the cervicovaginal DNA virome and understand how the virome may play a role in cervical cancer.

## RESULTS

### Cohort demographics and virome sequencing.

We conducted virome metagenomic next-generation sequencing of 28 CVL specimens from a subset of a cohort assembled by Łaniewski et al. (10). This cohort included nonpregnant, premenopausal, women from the Phoenix, AZ, metropolitan area and was used previously to study the relationship between cervicovaginal microbiota (16S rRNA gene sequencing) and cervical carcinogenesis. Five samples were dropped due to low viral read count (<50). Among the remaining 23 patients, 78.3% were HPV positive (Table 1). The majority (73.9%) showed no signs of cervical disease, while the remaining 26% had either cervical dysplasia or cervical cancer. Vaginal microbiomes were dominated by *Lactobacillus* (>80% *Lactobacillus* relative abundance) in 60.9% of the study participants and by non-*Lactobacillus* organisms in the remaining 39.1%. There was no evidence of genital inflammation in 65.2% of the women, while 34.8% had elevated levels of one or more inflammatory cytokines used to generate genital inflammatory scores. Of the study participants, 17.4% had a low vaginal pH (≤4.5), 47.8% had an intermediate vaginal pH (5.0), and 34.8% had a high vaginal pH (>5.0). The mean age of the women in this study was 38.1 years, and the average body mass index (BMI) was 29.2.

**TABLE 1:** Patient demographics and characteristics (*n* = 23)

Characteristic	Value
Age [mean ± SD (range)]	38.1 ± 8.6 (23–50)
Race and ethnicity [no. (%)]	
Non-Hispanic white	18 (78.3)
Hispanic	3 (13.0)
Other	2 (8.7)
BMI [mean ± SD (range)]	29.2 ± 10.1 (19–64)
BMI *n* (%)	
Normal (18.5–24.9)	10 (43.5)
Overweight (25.0–29.9)	5 (21.7)
Obese (30.0–34.9)	2 (8.7)
Extremely obese (≥35.0)	6 (26.1)
Disease status *n* (%)	
Healthy control	17 (73.9)
Cervical dysplasia	3 (13.0)
Cervical cancer	3 (13.0)
HPV status *n* (%)	
HPV negative	5 (21.7)
HPV positive	18 (78.3)
Vaginal pH [median (range)]	5.0 (4.5–7.5)
No. (%) with vaginal pH	
Low (≤4.5)	4 (17.4)
Intermediate (5.0)	11 (47.8)
High (>5.0)	8 (34.8)
No. (%) with vaginal microbiota	
* Lactobacillus* dominant (>80%)	14 (60.9)
Non-*Lactobacillus* dominant (<80%)	9 (39.1)
Genital inflammation [no. (%)]	
No	15 (65.2)
Yes	8 (34.8)

**FIG 1:**
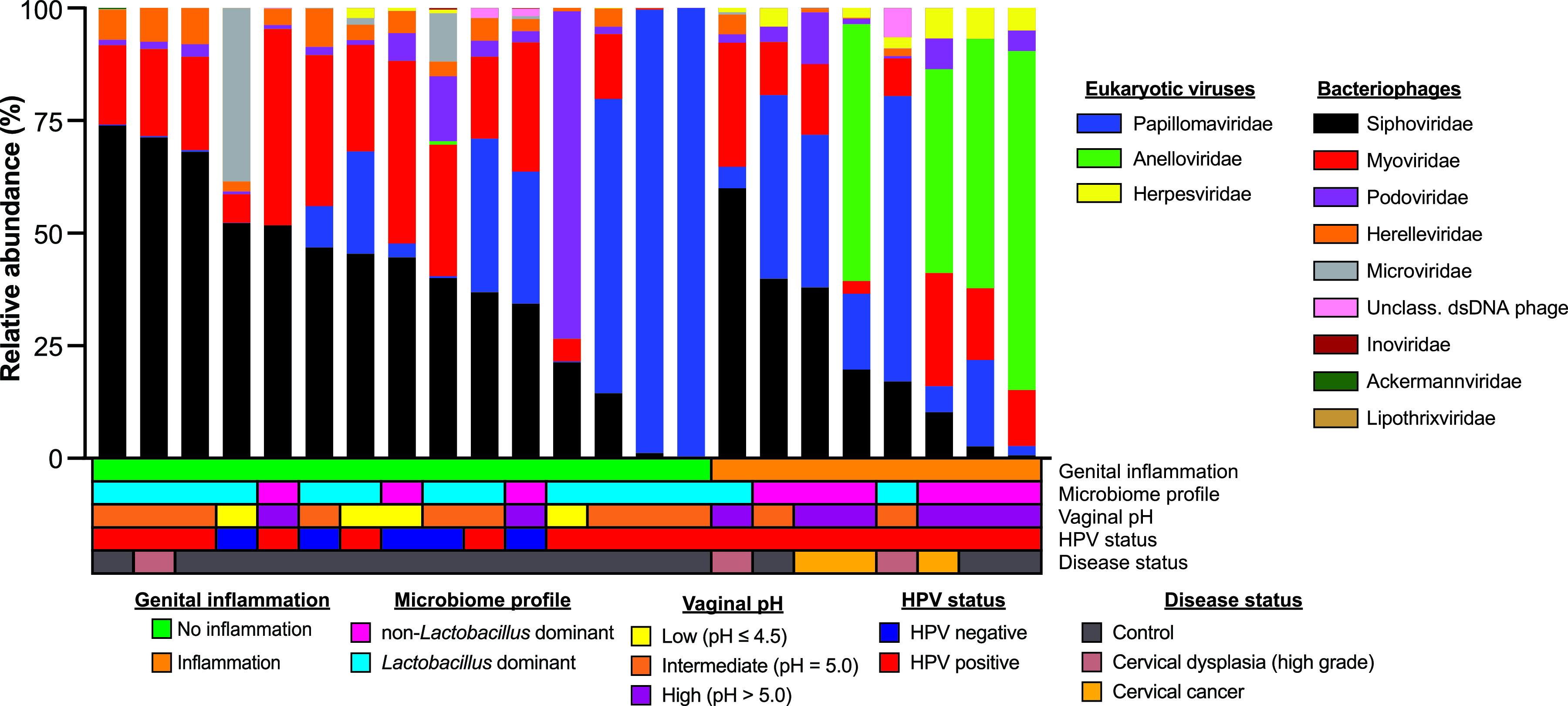
Cohort virome composition includes bacteriophages and anelloviruses. Relative abundance plot by viral family. Genital inflammation refers to the presence of proinflammatory cytokines, and microbiome profile is defined by *Lactobacillus* relative abundance. Low vaginal pH was defined as less than or equal to 4.5, intermediate pH was defined as equal to pH 5.0, and high pH was defined as greater than 5.0. HPV status was determined with a Roche linear array HPV genotyping assay, and disease status refers to cervical dysplasia, cervical cancer, or no cervical disease (controls).

We obtained a total of 19.1 million raw read pairs from 23 CVL samples, resulting in a median sequencing depth of 824,091 read pairs (standard deviation [SD], 137,249) per sample. After quality filtering and removal of reads mapping to the human genome, we obtained a total of 7.4 million reads, with a median sequencing depth of 195,992 reads (SD, 296,352). The most abundant viral families detected were *Siphoviridae*, *Papillomaviridae*, and *Myoviridae*, with *Siphoviridae* and *Myoviridae* being present in all samples (Fig. 1). *Anelloviridae* was also highly abundant in 50% (4/8) of the samples from patients with genital inflammation.

### Genital inflammation associated with lower alpha diversity and *Anelloviridae* species.

**FIG 2:**
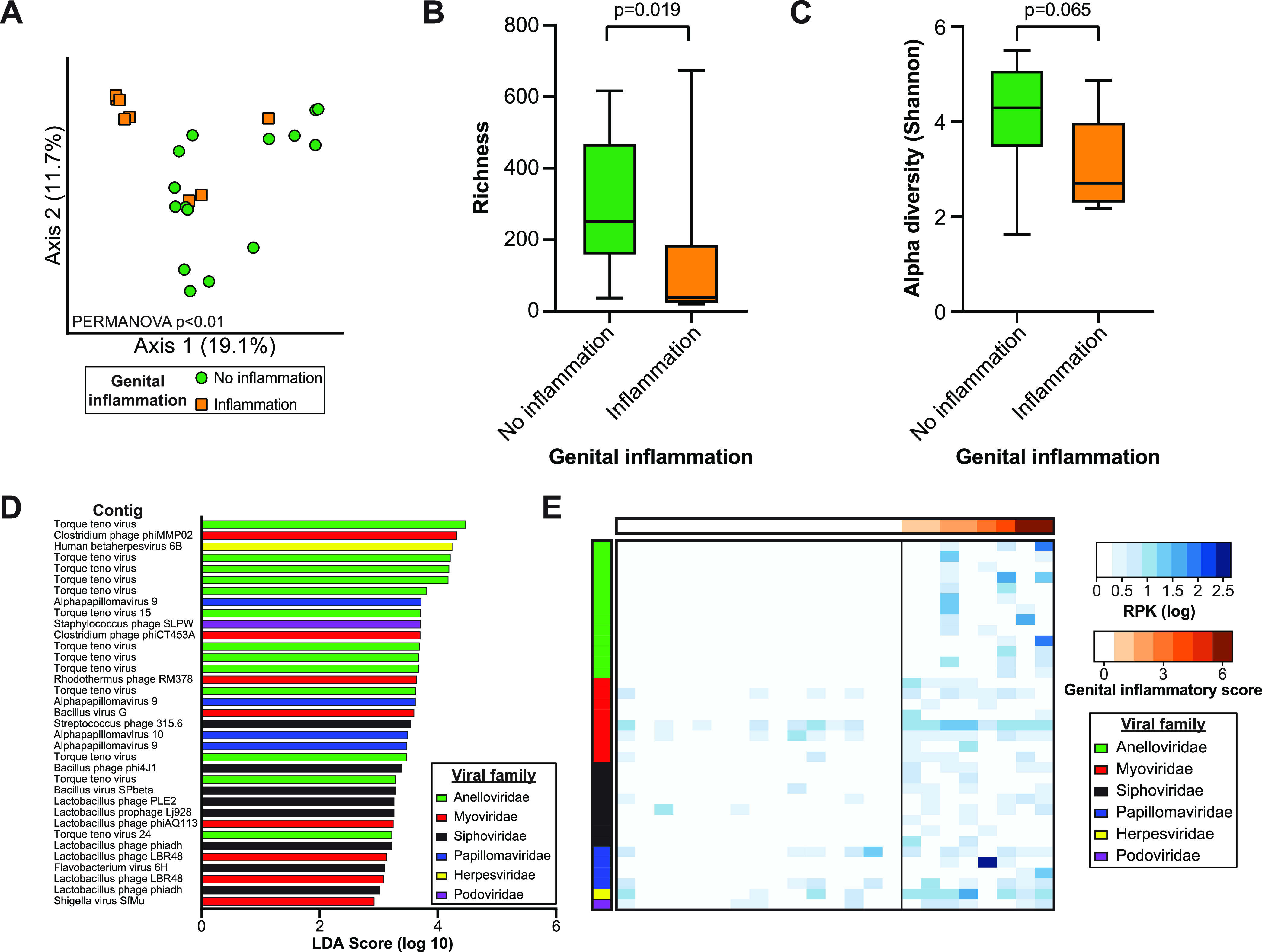
Alterations in cervicovaginal virome are associated with genital inflammation. (A) PCoA plot based on Bray-Curtis distances. Samples are colored by genital inflammation group. Statistical significance was assessed by PERMANOVA. (B) Richness of viral contig species in the no-inflammation (*n* = 15) and inflammation (*n* = 8) groups. Statistical significance was assessed by Mann-Whitney test. (C) Alpha diversity of viral contigs in the genital inflammation groups. Statistical significance was assessed by Mann-Whitney test. (D) Discriminant contigs between inflammation groups (LEfSe). (E) Abundance heat map showing log-transformed RPK counts of viruses associated with inflammation. Samples are ordered by increasing genital inflammatory score, and rows are grouped by viral family.

Since genital inflammation is a known risk factor for cervical carcinoma, we investigated the relationship between genital inflammation and the cervicovaginal virome. Genital inflammation was scored by a multiplex cytokine assay (see Materials and Methods) and classified into two categories: no inflammation (*n* = 15) or inflammation (*n* = 8) (Table 1). Principal coordinate analyses (PCoA) of unweighted Bray-Curtis dissimilarity showed that virome communities clustered separately by genital inflammation status (permutational multivariate analysis of variance [PERMANOVA], *P* = 0.002, *R*^2^ = 0.07) (Fig. 2A). Richness was significantly higher in samples with no inflammation (Mann-Whitney test, *P* = 0.019) (Fig. 2B), with a similar trend in Shannon diversity (Mann-Whitney test, *P* = 0.065) (Fig. 2C). We next sought to identify specific viruses associated with genital inflammation using linear discriminant analysis effect size (LEfSe) analyses. We identified 35 contigs associated with genital inflammation, including multiple *Anelloviridae* and bacteriophage contigs (Fig. 2D; Table S1). A heat map analysis showed that discriminant viruses were more abundant and prevalent in samples with inflammation (Fig. 2E). Anelloviruses were notably absent from all but one of the samples with no inflammation, indicating a difference between the inflammation groups. Taken together, these findings indicate that genital inflammation is associated with alterations in the cervicovaginal virome.

10.1128/msystems.00064-22.4 TABLE S1 Contigs associated with genital inflammation. Download Table S1, DOCX file, 0.02 MB. Copyright © 2022 Kaelin et al. 2022 Kaelin et al. https://creativecommons.org/licenses/by/4.0/ This content is distributed under the terms of the Creative Commons Attribution 4.0 International license.

### Anelloviruses are highly abundant and prevalent with genital inflammation and non-*Lactobacillus*-dominated microbiomes.

**FIG 3:**
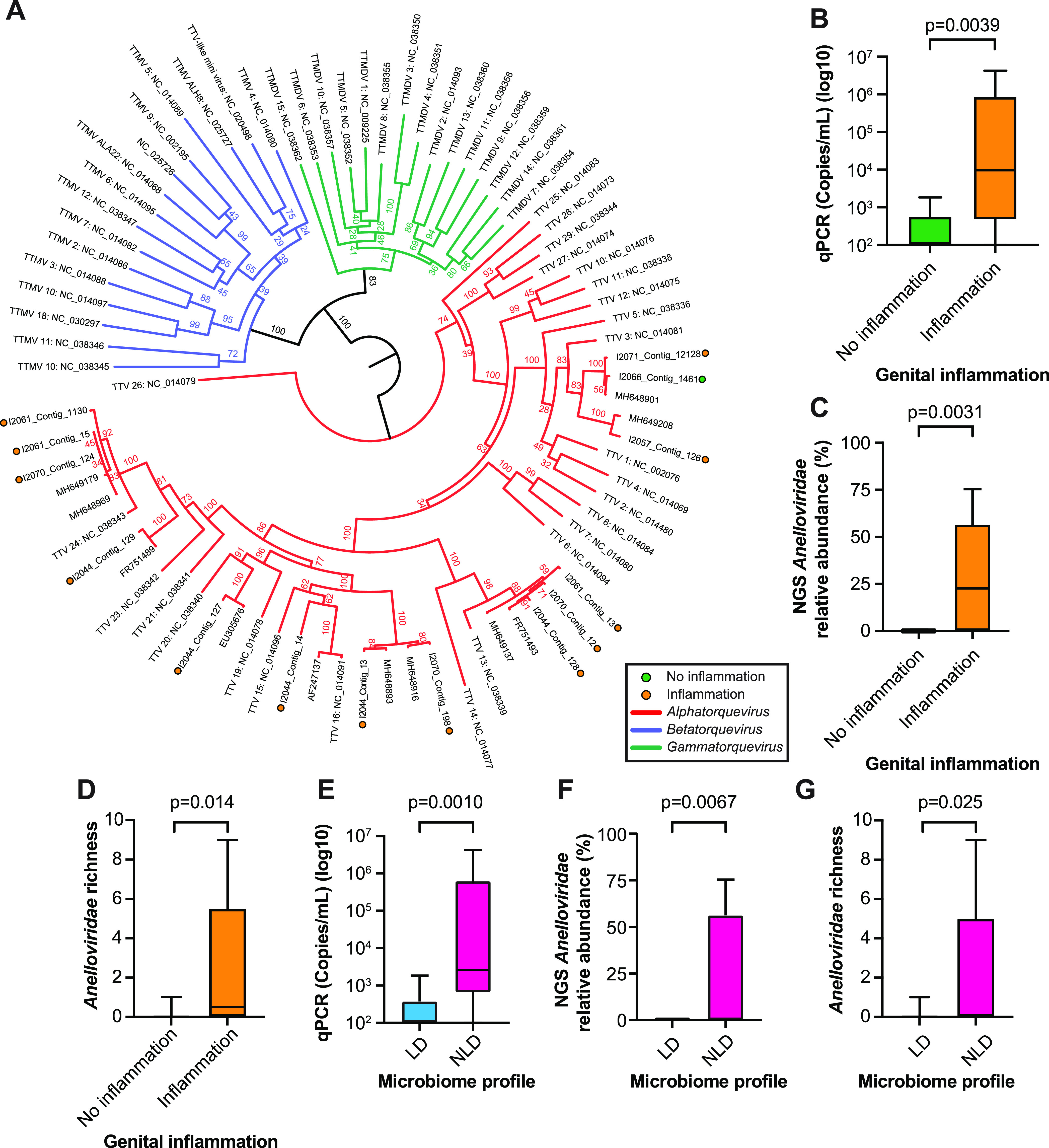
Anelloviruses are associated with genital inflammation. (A) *Anelloviridae* ORF1 amino acid contig phylogeny with *Alphatorquevirus*, *Betatorquevirus*, and *Gammatorquevirus* genera as references. Built with RAxML using PROTGAMMALG model and 1,000 bootstrap replicates. Colored circles indicate contigs built in this study. (B) *Alphatorquevirus* qPCR copy number comparison between genital inflammation groups. (C) NGS *Anelloviridae* relative abundance comparison between genital inflammation groups. (D) *Anelloviridae* contig richness by inflammation group. (E) *Alphatorquevirus* qPCR copy number comparison between microbiome profiles (*Lactobacillus* dominated and non-*Lactobacillus* dominated). (F) NGS *Anelloviridae* relative abundance between microbiome profiles. (G) *Anelloviridae* richness by microbiome profile. Statistical significance was assessed by Mann-Whitney test.

We conducted phylogenetic analysis to characterize the *Anelloviridae* species present in this cohort. All 14 *Anelloviridae* contigs belonged to the *Alphatorquevirus* genus, with 13 of the 14 contigs being associated with inflammation (Fig. 3A). Since next-generation sequencing (NGS) data indicated that anelloviruses were associated with genital inflammation, we performed additional molecular validation by quantitative PCR (qPCR) to compare *Alphatorquevirus* viral loads. Notably, *Alphatorquevirus* genome copy numbers were significantly increased in the inflammation group (Mann-Whitney test, *P* = 0.0039) (Fig. 3B). This qPCR result is consistent with NGS *Anelloviridae* relative abundance being higher in samples with inflammation (Mann-Whitney test, *P* = 0.0031) (Fig. 3C). To understand whether the number of *Anelloviridae* species differed between groups, we compared contig richness. We found that the inflammation group had significantly more *Anelloviridae* species than the no-inflammation group (Mann-Whitney test, *P* = 0.014) (Fig. 3D). We observed similar results when analyzing anellovirus levels by bacterial microbiome profile. Anellovirus genome copy numbers, relative abundance, and richness were higher in the non-*Lactobacillus*-dominated microbiomes (Mann-Whitney test, *P* = 0.0010, *P* = 0.0067, and *P* = 0.025, respectively) (Fig. 3E to G). These results further support the conclusion that *Anelloviridae* species are associated with a dysbiotic cervicovaginal microbiome and genital inflammation.

### HPV infection is not associated with genital inflammation.

To determine if HPV infection was associated with specific features of the local microenvironment in this cohort, we examined the HPV contigs generated. The majority of HPV contigs called were from the genus *Alphapapillomavirus* (Fig. S2A). We validated the HPV contig analysis with results from the Roche linear array HPV genotyping assay (10) and found that there was 100% concordance (14 of 14) in HPV types detectable by linear array assay. We found no significant difference in HPV contig richness by genital inflammation status or microbiome profile (Fig. S2B and C). This analysis supports the hypothesis that virome alterations may better explain changes in genital inflammation and vaginal microbiome profile.

10.1128/msystems.00064-22.2 FIG S2 Papillomavirus phylogenetic analysis. (A) Phylogeny of papillomavirus genera associated with human infection. Stars next to labels indicate contigs built in this study. Generated using an alignment of the E1 gene regions and built using RAxML and 1,000 bootstrap replicates. (B) Richness of papillomavirus contigs between inflammation groups. Statistical significance was assessed by Mann-Whitney test. (C) Richness of papillomavirus contigs between microbiome profiles. Statistical significance was assessed by Mann-Whitney test. Download FIG S2, EPS file, 2.2 MB. Copyright © 2022 Kaelin et al. 2022 Kaelin et al. https://creativecommons.org/licenses/by/4.0/ This content is distributed under the terms of the Creative Commons Attribution 4.0 International license.

### Bacteriophage contigs are associated with bacterial microbiome profiles.

**FIG 4:**
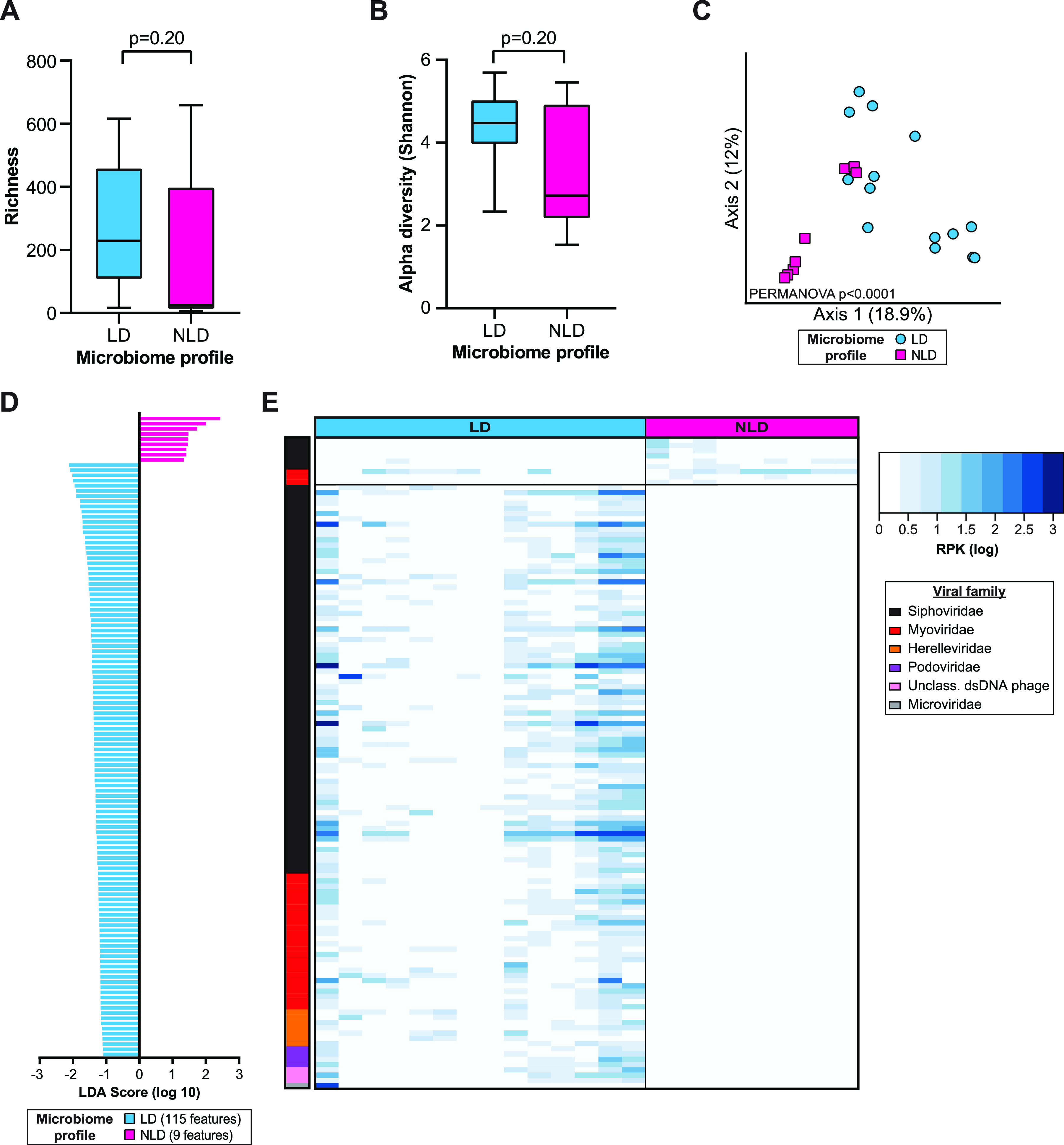
Bacteriophages are closely associated with *Lactobacillus* microbiome profile differences. (A) Richness of viral bacteriophage contigs in *Lactobacillus*-dominated (LD) and non-*Lactobacillus*-dominated (NLD) microbiome profiles. Statistical significance was assessed by Mann-Whitney test. (B) Alpha diversity by bacterial microbiome profile. Statistical significance was assessed by Mann-Whitney test. (C) PCoA of Bray-Curtis distances colored by bacterial microbiome profile. Statistical significance was assessed using PERMANOVA. (D) Bar plot showing linear discriminant analysis (LDA) scores of contigs discriminating between microbiome profiles identified by LEfSe. (E) Abundance heat map of log-transformed RPK counts of LEfSe discriminant contigs (rows) across all samples (columns).

To better understand the bacteriophages present in this cohort, we performed a separate analysis considering only bacteriophages. Since the healthy vaginal microbiome is dominated by *Lactobacillus* species, we hypothesized that cervicovaginal virome profiles may be associated with bacterial microbiome composition. There were no differences in virome richness and alpha diversity measures between *Lactobacillus*-dominated and non-*Lactobacillus*-dominated microbiome profiles (Fig. 4A and B). However, PCoA of unweighted Bray-Curtis dissimilarity showed that samples clustered separately by microbiome profile (PERMANOVA, *P* < 0.0001, *R*^2^ = 0.1) (Fig. 4C). We next used LEfSe to help explain the clustering observed in the PCoA and identify discriminant bacteriophage contigs between the *Lactobacillus*-dominated and non-*Lactobacillus*-dominated microbiome profiles. LEfSe identified 115 discriminating bacteriophage contigs associated with the *Lactobacillus*-dominated group and 9 contigs associated with the non-*Lactobacillus*-dominated group (Fig. 4D; Table S2). The most common discriminant bacteriophage family identified was *Siphoviridae*, with the most common host being bacteria belonging to the genus *Bacillus*. *Lactobacillus* bacteriophages were associated only with the *Lactobacillus*-dominated group. We plotted counts for these features on a heat map by family to observe feature distribution across samples (Fig. 4E). *Siphoviridae* was the most prevalent feature, followed by *Myoviridae* and *Herelleviridae*. These analyses demonstrate alterations in the bacteriophage virome and distinct differences in the bacteriophage community composition when compared by bacterial microbiome profiles.

10.1128/msystems.00064-22.5 TABLE S2 Contigs associated with *Lactobacillus*-dominated microbiomes. Download Table S2, DOCX file, 0.02 MB. Copyright © 2022 Kaelin et al. 2022 Kaelin et al. https://creativecommons.org/licenses/by/4.0/ This content is distributed under the terms of the Creative Commons Attribution 4.0 International license.

### Patients with genital inflammation show distinct bacteria-bacteriophage transkingdom interactions.

**FIG 5:**
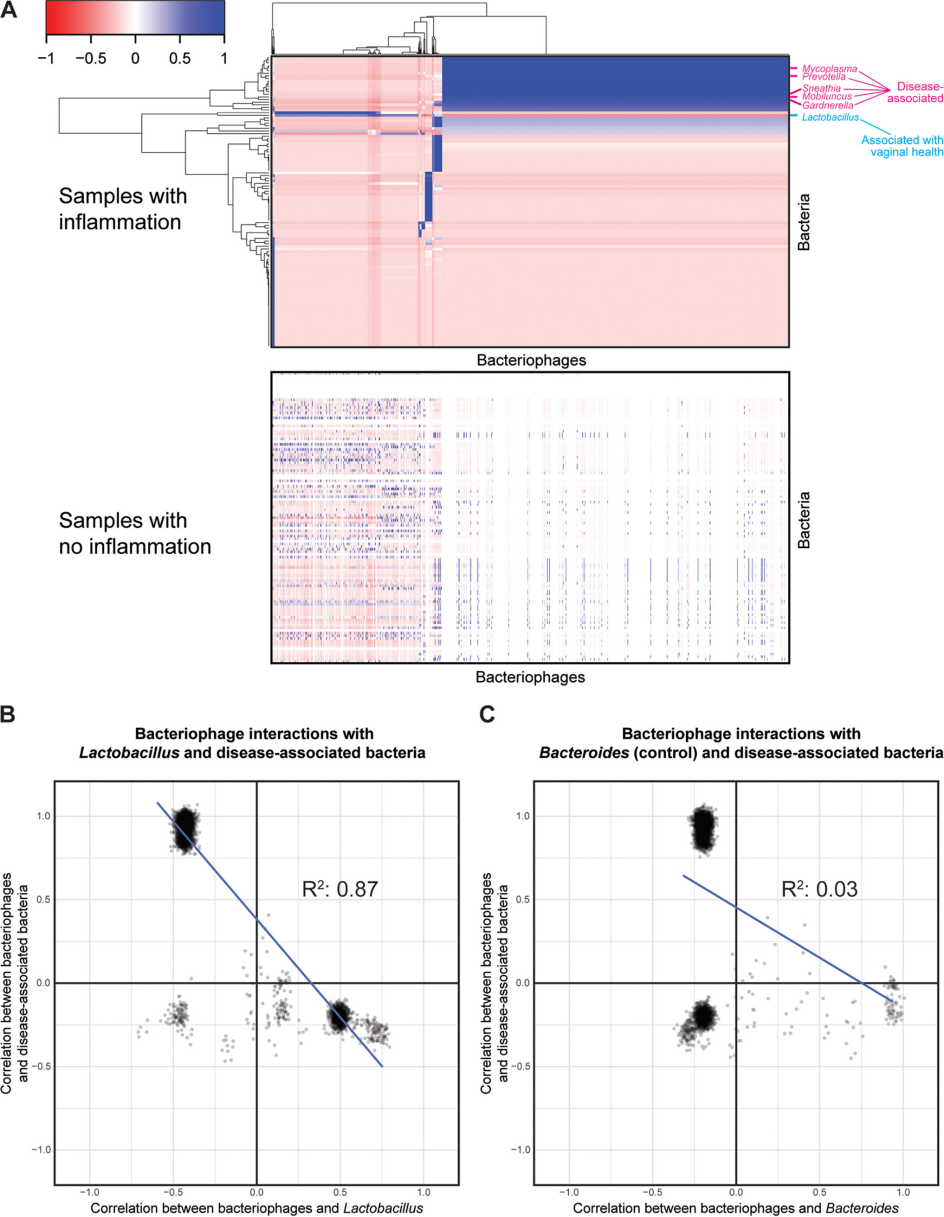
Transkingdom interactions between bacteriophages and bacteria are altered by genital inflammation status. (A) Hierarchical clustered heat maps of Pearson correlation of bacterial genera and bacteriophages in samples with inflammation (top heat map) and samples with no inflammation (bottom heat map). Clustering was performed on inflammation samples, with row and column order being maintained for the noninflammation sample correlation heat map. (B) Relationship between bacteriophage correlation with *Lactobacillus* (*x* axis) and bacteriophage correlation with BV-associated bacteria (*Mobiluncus*, *Mycoplasma*, *Gardnerella*, *Prevotella*, *and Sneathia*; *y* axis). (C) Control scatterplot of relationship between bacteriophage correlation with *Bacteroides* (*x* axis) and bacteriophage correlation with BV-associated bacteria (*y* axis).

We next integrated bacterial relative abundance data with the bacteriophage data to characterize transkingdom interactions between the bacterial microbiome and the virome. Bacterial microbiome sequencing analysis using data from a prior study of this cohort (10) showed that the vaginal microbiome was dominated primarily by *Lactobacillales*. The orders *Bifidobacteriales*, *Clostridiales*, *Coriobacteriales*, *Bacteroidales*, *Fusobacteriales*, and *Mycoplasmatales* were also present in lower abundances in vaginal samples (Fig. S3). Therefore, we hypothesized that specific bacteria-bacteriophage interactions were associated with features of the microenvironment linked with HPV persistence and progression to cervical cancer. Clustered heat map analysis of Pearson correlation showed that samples with genital inflammation had distinct interactions that were absent in samples with no inflammation (Fig. 5A). The genera *Mobiluncus*, *Mycoplasma*, *Gardnerella*, *Prevotella*, and *Sneathia* (bacteria associated with BV, HPV infection, and cervical cancer progression [6, 10, 15]) were positively correlated with specific bacteriophages (Fig. 5A, top heat map; Data Set S1). In contrast, these bacteriophage interactions were inversely correlated with *Lactobacillus* (bacteria associated with healthy microbiomes) (*R*^2^ = 0.87) (Fig. 5A and B). As a control, we compared *Bacteroides*, which is not commonly associated with pathogenesis. Transkingdom interactions with *Bacteroides* were independent from those with BV-associated bacteria (*R*^2^ = 0.03) (Fig. 5C). Hence, this suggests that there are distinct transkingdom interactions associated with genital inflammation.

10.1128/msystems.00064-22.3 FIG S3 Bacterial relative abundance of the cohort, grouped by the 10 most abundant bacterial orders. Download FIG S3, EPS file, 1.5 MB. Copyright © 2022 Kaelin et al. 2022 Kaelin et al. https://creativecommons.org/licenses/by/4.0/ This content is distributed under the terms of the Creative Commons Attribution 4.0 International license.

10.1128/msystems.00064-22.6 DATA SET S1 Pearson correlation matrix of bacteria and bacteriophages in samples with inflammation shown in Fig. 5A. Download Data Set S1, XLSX file, 0.4 MB. Copyright © 2022 Kaelin et al. 2022 Kaelin et al. https://creativecommons.org/licenses/by/4.0/ This content is distributed under the terms of the Creative Commons Attribution 4.0 International license.

## DISCUSSION

Previous studies have established a link between the vaginal bacterial microbiome and cervical cancer (10, 14, 19, 27–30). For example, low *Lactobacillus* relative abundance has been associated with both HPV infection and progression of cervical neoplasm. Other features of the local microenvironment that have been associated with cervical cancer include inflammation and elevated vaginal pH (10). Despite the well-established association between bacterial microbiome and cervical carcinogenesis, the relationship between the cervicovaginal virome and cervical cancer has not previously been investigated.

In this study of the cervicovaginal DNA virome of women with and without HPV infection, we identified associations between the virome and features of the local microenvironment associated with cervical carcinogenesis, i.e., bacterial microbiome and inflammation. We found that anelloviruses were associated with genital inflammation. Our data indicate that transkingdom interactions were associated with microbiome profiles (*Lactobacillus* dominated versus non-*Lactobacillus* dominated) and genital inflammation. These findings suggest that the cervicovaginal virome may be implicated in microbiome changes and inflammation, which can contribute to HPV persistence and progression to cervical cancer.

Chronic inflammation is associated with cancer development and progression (9). Genital inflammation has been associated with increased likelihood of progression to cervical carcinoma, as well as increased risk of HIV acquisition and other sexually transmitted infections (10, 31). We found that virome profiles differed significantly when categorized by genital inflammation. Samples from women with genital inflammation were associated with decreased viral species richness and clustered separately from samples with no evidence of inflammation. The alterations between the virome and genital inflammation are consistent with previous findings on changes in the bacterial microbiome (10). Furthermore, this is consistent with previous findings linking the virome with inflammation in the gut (23, 32).

We found that anelloviruses, including torque teno viruses (TTVs), were associated with genital inflammation. These results were consistent between NGS (which determine viral relative abundance) and qPCR assays (which determine viral load). Human TTVs, small circular single-strand-DNA (ssDNA) viruses belonging to the family *Anelloviridae*, have been observed in various proportions of the population around the world, up to >90% in some studies (33). Anelloviruses can induce proinflammatory cytokine responses (34), which may also contribute to genital inflammation. Increased *Anelloviridae* contig richness in samples with inflammation in our study indicates multiple *Anelloviridae* coinfections, which is consistent with prior findings in serum (35). Interestingly, one study found that coinfection with TTV and HPV was associated with increased tumor progression in patients treated for laryngeal carcinoma (36). TTV infections have also been observed in women with vaginitis (37). Similarly, immunosuppression status has been associated with plasma anellovirus levels, further suggesting a link between anelloviruses and host immunity (38).

The presence of *Lactobacillus* species in the vaginal bacterial microbiome has been established as a protective agent against HPV infection, viral persistence, and progression of cervical neoplasm (10, 39–42). Therefore, we investigated the relationship between the virome and microbiome community states, defined by *Lactobacillus* species abundance. We found that *Lactobacillus* bacteriophages were only associated with the *Lactobacillus*-dominated microbiome. While many bacteriophages were identified to be associated with the *Lactobacillus*-dominated microbiome, relatively few were identified as associated with the non-*Lactobacillus*-dominated microbiome. This may be due to the more heterogeneous nature of the non-*Lactobacillus*-dominated microbiomes compared to the *Lactobacillus*-dominated microbiomes. Transkingdom analyses between the bacterial microbiome and bacteriophages revealed that women with genital inflammation and BV-associated bacteria (such as *Mobiluncus*, *Mycoplasma*, *Gardnerella*, *Prevotella*, and *Sneathia*) shared similar interaction profiles. This is consistent with findings that BV-associated genera overlapped with those associated with cervical carcinoma (6, 10, 15). In contrast, these interactions were absent from women with no genital inflammation.

To our knowledge, this is the first study to investigate the relationship between the cervicovaginal virome and local microenvironmental factors associated with HPV and cervical cancer. One limitation of this study is the small sample size. Hence, studies are needed to establish the link between genital inflammation and the virome, particularly in the context of inflammatory diseases in women’s health.

In conclusion, we identified associations between cervicovaginal DNA virome composition and bacterial microbiome and genital inflammation. Both vaginal *Lactobacillus* relative abundance and genital inflammation were associated with changes in the cervicovaginal virome. *Anelloviridae* species, including *Alphatorquevirus* species, were also associated with genital inflammation. Additionally, we observed distinct bacteria-bacteriophage transkingdom interactions between established disease associated bacteria and bacteriophages. Overall, these findings indicate that the cervicovaginal virome may play a role in the HPV-mediated persistence and progression to cervical cancer and is an important area of future research.

## MATERIALS AND METHODS

### Study population and specimen collection.

A subset of 28 premenopausal, nonpregnant women was taken from a study by Łaniewski et al. (10). Patients were recruited at the University of Arizona Cancer Center in Phoenix, AZ, in accordance with federal guidelines and regulations and the Declaration of Helsinki. All patients provided written consent, and all research and related activities involving human subjects were approved by the University of Arizona and Arizona State University Institutional Review Boards (10). Vaginal swabs and cervicovaginal lavage (CVL) samples were collected by a physician. The first vaginal swab was collected using an Eswab collection system with Amies transport medium (Copan Diagnostics) and used for HPV genotyping and 16S rRNA sequencing analyses. The second vaginal swab was collected using a sterile cotton swab and used for assessment of vaginal pH. CVL samples were collected using 10 mL of sterile 0.9% saline (Teknova) and used for multiplex cytokine and virome analyses. All collected samples were immediately placed on ice and frozen at −80°C until processing. CVL were thawed on ice, clarified by centrifugation (700 × *g* for 10 min at 4°C) and aliquoted (∼400 μL) to avoid multiple freeze-thaw cycles, and stored at −80°C prior to analyses.

### Cervicovaginal microbiome, HPV genotyping, and genital inflammation score.

Women were classified as HPV negative or positive. HPV status was determined with a linear array HPV genotyping test (Roche), which detects 37 anogenital HPV genotypes (6, 11, 16, 18, 26, 31, 33, 35, 39, 40, 42, 45, 51, 52, 53, 54, 55, 56, 58, 59, 61, 62, 64, 66, 67, 68, 69, 70, 71, 72, 73 [MM9], 81, 82 [MM4], 83 [MM7], 84 [MM8], IS39, and CP6108), including 13 high-risk types. DNA extracted from vaginal swabs was used in the assay (10). DNA was isolated using a PowerSoil DNA kit (MO BIO Laboratories), eluted with 50 μL of nuclease-free water, and stored at −80°C prior to analysis. 2.5 μL of DNA samples was used in the amplification PCR of an ∼450-bp fragment of the polymorphic L1 region of the HPV genome, followed by the hybridization and colorimetric detection steps as described in the manufacturer’s instruction. An amplicon of human β-globin gene was used as an internal control for extraction and amplification for each individually processed DNA sample.

Vaginal bacterial microbiome analysis was performed previously by 16S rRNA gene sequencing using DNA extracted from vaginal swabs (10). Amplicon library preparation and 16S rRNA sequencing were performed by Second Genome, Inc. The V4 region of bacterial 16S rRNA gene was amplified from the genomic DNA using fusion primers and sequenced for 250 cycles on the MiSeq platform (Illumina). The samples were analyzed using USEARCH and the Greengenes reference database to generate the abundance table for operational taxonomic units. Vaginal *Lactobacillus* relative abundance was recorded as less than 80% (for non-*Lactobacillus*-dominated profiles) or greater than 80% (for *Lactobacillus*-dominated profiles).

A multiplex cytokine assay for interleukin 1α (IL-1α), IL-1β, IL-8 (CXCL8), MIP-1β (CCL4), MIP-3α (CCL20), RANTES (CCL5), and tumor necrosis factor alpha (TNF-α) (Millipore) was performed on CVL samples (100 μL) using Milliplex MAP human cytokine/chemokine and Th17 magnetic bead panels (Millipore) in accordance with the manufacturer’s instructions. Data were collected with a Bio-Plex 200 instrument and analyzed using Manager 5.0 software (Bio-Rad). The concentrations were calculated using four-parameter logistic regression curve fit. All samples were assayed in duplicate. The log transformation was applied to normalize the data. To determine genital inflammation status, patients were assigned one point for each of seven tested cytokines when the level was in the upper quartile (10). In our previous study, we showed that women with cervical cancer exhibited significantly elevated inflammatory scores compared to HPV-negative women without cervical dysplasia (10). In addition, other clinical studies investigating the vaginal microbiome also utilized this method as a proxy for genital inflammation (43–45). Women with genital inflammatory scores of 0 were classified as having no genital inflammation, and women with genital inflammatory scores of 1 or greater were classified as having evidence of genital inflammation.

### Total nucleic acid extraction and next-generation sequencing.

CVL specimens were brought to a volume of 800 μL with phosphate-buffered saline (PBS) (∼300 to 500 μL of PBS was added to each). Specimens were vortexed and centrifuged at 13,500 rpm for 10 min. The supernatant was collected, and total nucleic acid was extracted using the eMAG instrument (bioMérieux). PBS negative controls were also included in the same extraction process to assess contamination. The PBS negative extraction controls were spiked with Escherichia virus lambda DNA prior to DNA amplification. DNA was amplified with GenomiPhi V2 (GE Healthcare) before Illumina library construction. Libraries were constructed using the Kapa Biosystems Hyperplus kit in accordance with the manufacturer’s protocol. Briefly, DNA was enzymatically sheared, end repaired and A tailed, adapter ligated, and amplified prior to pooling and sequencing. Samples and spiked PBS negative controls were pooled for sequencing in four Illumina MiSeq v2 2 × 250 sequencing runs. Five samples were excluded from the study due to low (<50) viral read counts, resulting in 23 samples being analyzed.

### Virome analysis.

Adapter trimming and quality filtering of sequencing data were conducted with BBTools (46) (BBDuk with the parameters k = 25 hdist = 1 ktrim=r qtrim=rl mink = 11 trimq = 30 minlength = 75 minavgquality = 20 removeifeitherbad=f otm=t tpe=t overwrite=t). PhiX reads (BBDuk with the parameters k = 31 hdist = 1 overwrite=t) and reads matching the human genome (BBMap with the parameters minid = 0.95 maxindel = 3 bwr = 0.16 bw = 12 quickmatch fast minhits = 2) were removed. Paired reads were merged and deduplicated (two rounds of deduplication, with minidentity of 99 before merging and 100 after merging). Contig assembly was performed using sequencing reads from each sample with IDBA-UD (version 1.1.0) (47). Contigs shorter than 500 nucleotides were filtered out using BBTools and deduplicated at a minimum identity of 99 (48). BWA-MEM was then used with the parameters -L 97, 97, and -M to map the QC reads to the previously built contig database (version 0.7.17) (49). To identify viral contigs, BLASTx was performed on all contigs against the RefSeq viral database (minimum E value 1e−3, maximum target sequences = 1). Additionally, contigs were queried with VirSorter to identify bacteriophage contigs (50). Bacteriophage analyses were based on bacteriophage contigs identified either by BLASTx or VirSorter.

To assess the nonviral portion of the sequencing data, nonviral contigs/reads were subsequently queried against the NCBI nucleotide database (downloaded June 2020) using Megablast (min E value 1e−10) and assigned based on the top BLAST hit. Collectively, the fraction of sequencing data mappable to humans, viruses, bacteria, and other (gorilla, tapeworm, yeast, etc.) and those that were unmapped is shown in Fig. S1A. Log-transformed RPK (reads per kilobase) counts were determined by normalizing to 55,000 read depth and contig length. Decontam (51) was used with a threshold of 0.1 to identify and remove contaminants by comparing samples to extraction controls (Fig. S1B). Contaminants were primarily classified as Escherichia virus lambda (4 samples), with a smaller relative abundance of Stx2-converting phage 86 (1 sample) and Campylobacter virus CP21 (controls only) (Fig. S1C). RPK counts less than 1 were masked.

10.1128/msystems.00064-22.1 FIG S1 Sequencing data and contamination analysis. (A) Fraction of mappable sequencing data. (B) Relative abundance of viral counts removed by decontam. Red indicates counts removed as contaminants. (C) Species classification of viral counts removed as contaminants. Download FIG S1, EPS file, 2.1 MB. Copyright © 2022 Kaelin et al. 2022 Kaelin et al. https://creativecommons.org/licenses/by/4.0/ This content is distributed under the terms of the Creative Commons Attribution 4.0 International license.

Shannon and Bray-Curtis diversity measurements were performed using the vegan and ape R packages. PCoA analysis was conducted using phyloseq (52). LEfSe analysis was performed using the online Galaxy module (53). Pearson correlation between bacterial and bacteriophage abundance was calculated in R using corrplot (54). Statistical significance for continuous variables was determined using the Mann-Whitney U test. Statistical significance for principal coordinate analyses was determined by PERMANOVA testing. Heat maps were plotted using gplots, and all other graphs were made in GraphPad Prism.

### ATV qPCR.

Three microliters of total nucleic acid input was used with the *Alphatorquevirus* (ATV) forward primer (5′-GTC CCG IAG GTG AGT TTA-3′), reverse primer (5′-AGC CCG GCC AGT CC-3′), and probe (5′–6-carboxyfluorescein [6-FAM]–TCA AGG GGC AAT TCG GGC T–tetramethylrhodamine [TAMRA]–3′) at a 10 μM concentration with ABI TaqMan 2× fast universal PCR master mix. Cycling conditions were 95°C for 20 s followed by 40 cycles of 95°C for 1 s and 60°C for 20 s.

### Phylogenetic analysis.

The *Anelloviridae* phylogeny (Fig. 3A) was an amino acid alignment of the ORF1 gene of all NCBI RefSeq *Alphatorquevirus*, *Betatorquevirus*, and *Gammatorquevirus* genomes against the contig ORF1 regions using MUSCLE v3.8.425 (55). RAxML v8.2.12 was used to generate a phylogenetic tree using the PROTGAMMALG substitution model with 1,000 bootstrap replicates (56). Tree label editing and rooting were completed using FigTree v1.4.4 (57).

The HPV phylogeny (Fig. S2A) was generated by aligning the E1 genes of NCBI RefSeq *Alphapapillomavirus*, *Betapapillomavirus*, *Gammapapillomavirus*, and *Mupapillomavirus* genomes against the E1 regions of the HPV contigs using MUSCLE. RAxML was used to generate a phylogenetic tree using the GTRGAMMA model with 1,000 bootstrap replicates. Tree label editing and rooting were conducted using FigTree.

### Data availability.

Sequencing data have been deposited in the NCBI Sequence Read Archive under BioProject accession number PRJNA749321. Code used for reproduction of this analysis is available at https://github.com/ASU-Lim-Lab/CVL_UofA.
