# Supplementary material for: Cervicovaginal DNA Virome Alterations Are Associated with Genital Inflammation and Microbiota Composition
Source: mSystems. 2022 Mar 28;7(2):e00064-22. doi: 10.1128/msystems.00064-22 (PMC9040584; doi:10.1128/msystems.00064-22)
Supplement: TABLE S1 [file msystems.00064-22-st001.docx]

**Supplementary Table 1. Contigs associated with genital inflammation.**

| **Contig name** | **LDA score (log10)** | **p value** | **Family** | **Species** |
| --- | --- | --- | --- | --- |
| I2061_Contig_1123 | 4.18939814 | 0.01312757 | Anelloviridae | Torque teno virus |
| I2044_Contig_128 | 3.625527235 | 0.04770198 | Anelloviridae | Torque teno virus |
| I2071_Contig_11785 | 3.282002401 | 0.04770198 | Anelloviridae | Torque teno virus |
| I2070_Contig_12 | 4.214823286 | 0.01312757 | Anelloviridae | Torque teno virus |
| I2070_Contig_198 | 3.710110562 | 0.04770198 | Anelloviridae | Torque teno virus 15 |
| I2044_Contig_13 | 3.816521781 | 0.04770198 | Anelloviridae | Torque teno virus |
| I2044_Contig_129 | 3.667575033 | 0.04770198 | Anelloviridae | Torque teno virus |
| I2057_Contig_126 | 4.476342083 | 0.04770198 | Anelloviridae | Torque teno virus |
| I2071_Contig_12128 | 3.469447874 | 0.04770198 | Anelloviridae | Torque teno virus |
| I2061_Contig_13 | 4.176715899 | 0.04770198 | Anelloviridae | Torque teno virus |
| I2070_Contig_124 | 3.669495388 | 0.04770198 | Anelloviridae | Torque teno virus |
| I2044_Contig_116 | 3.688835541 | 0.01312757 | Anelloviridae | Torque teno virus |
| I2070_Contig_176 | 3.219341744 | 0.04770198 | Anelloviridae | Torque teno virus 24 |
| I2056_Contig_1237 | 3.244088498 | 0.00338762 | Myoviridae | Lactobacillus phage phiAQ113 |
| I2071_Contig_1338 | 3.640596499 | 0.02255768 | Myoviridae | Rhodothermus phage RM378 |
| I2071_Contig_12447 | 2.921844486 | 0.04770198 | Myoviridae | Shigella virus SfMu |
| I2056_Contig_1161 | 3.081699761 | 0.04770198 | Myoviridae | Lactobacillus phage LBR48 |
| I2071_Contig_13604 | 4.318927837 | 0.00084218 | Myoviridae | Clostridium phage phiMMP02 |
| I2065_Contig_1103 | 3.596922774 | 0.00637553 | Myoviridae | Bacillus virus G |
| I2059_Contig_1473 | 3.131099434 | 0.04770198 | Myoviridae | Lactobacillus phage LBR48 |
| I2066_Contig_1444 | 3.701508149 | 0.04493195 | Myoviridae | Clostridium phage phiCT453A |
| I2067_Contig_1852 | 3.386200383 | 0.00080005 | Siphoviridae | Bacillus phage phi4J1 |
| I2060_Contig_1213 | 3.012386265 | 0.04770198 | Siphoviridae | Lactobacillus phage phiadh |
| I2065_Contig_1600 | 3.262191434 | 0.02009378 | Siphoviridae | Lactobacillus phage PLE2 |
| I2068_Contig_183 | 3.538733888 | 0.04493195 | Siphoviridae | Streptococcus phage 315.6 |
| I2054_Contig_1460 | 3.277732497 | 0.0367217 | Siphoviridae | Bacillus virus SPbeta |
| I2060_Contig_1270 | 3.256641628 | 0.04770198 | Siphoviridae | Lactobacillus prophage Lj928 |
| I2071_Contig_1835 | 3.091294059 | 0.04770198 | Siphoviridae | Flavobacterium virus 6H |
| I2056_Contig_1280 | 3.216795988 | 0.04770198 | Siphoviridae | Lactobacillus phage phiadh |
| I2069_Contig_1148 | 3.621047248 | 0.01786558 | Papillomaviridae | Alphapapillomavirus 9 |
| I2071_Contig_11591 | 3.489940441 | 0.01312757 | Papillomaviridae | Alphapapillomavirus 10 |
| I2061_Contig_178 | 3.47950654 | 0.04770198 | Papillomaviridae | Alphapapillomavirus 9 |
| I2069_Contig_135 | 3.714661647 | 0.03666679 | Papillomaviridae | Alphapapillomavirus 9 |
| I2059_Contig_18 | 4.241485192 | 0.00072898 | Herpesviridae | Human betaherpesvirus 6B |
| I2054_Contig_11358 | 3.707493158 | 0.03055894 | Podoviridae | Staphylococcus phage SLPW |
