# Supplementary material for: Cervicovaginal DNA Virome Alterations Are Associated with Genital Inflammation and Microbiota Composition
Source: mSystems. 2022 Mar 28;7(2):e00064-22. doi: 10.1128/msystems.00064-22 (PMC9040584; doi:10.1128/msystems.00064-22)
Supplement: TABLE S2 [file msystems.00064-22-st002.docx]

**Supplementary Table 2.** **Contigs associated with Lactobacillus-dominant microbiome.**

| **Contig name** | **LDA score (log10)** | **p value** | **Family** | **Species** |
| --- | --- | --- | --- | --- |
| I2059_Contig_1400 | 4.24496807 | 0.00375949 | Siphoviridae | Paenibacillus virus Vegas |
| I2047_Contig_1754 | 4.16286848 | 0.00375949 | Siphoviridae | Staphylococcus prophage phiPV83 |
| I2054_Contig_11019 | 4.02834208 | 0.0276947 | Siphoviridae | Staphylococcus phage SPbeta-like |
| I2059_Contig_1325 | 4.00203621 | 0.00375949 | Siphoviridae | Staphylococcus prophage phiPV83 |
| I2056_Contig_1641 | 3.89726333 | 0.00375949 | Siphoviridae | Staphylococcus prophage phiPV83 |
| I2047_Contig_1361 | 3.81030545 | 0.00375949 | Siphoviridae | Paenibacillus virus Vegas |
| I2056_Contig_1147 | 3.80769717 | 0.00375949 | Siphoviridae | Paenibacillus virus Vegas |
| I2072_Contig_1264 | 3.5567394 | 0.0276947 | Myoviridae | Lactobacillus phage phiAQ113 |
| I2047_Contig_1667 | 3.55298322 | 0.00375949 | Siphoviridae | Bacillus phage PfEFR-4 |
| I2047_Contig_1621 | 3.48305324 | 0.01488788 | Siphoviridae | Staphylococcus virus 108PVL |
| I2054_Contig_11229 | 3.45274836 | 0.00375949 | Siphoviridae | Paenibacillus virus Vegas |
| I2059_Contig_1449 | 3.4217359 | 0.01488788 | Siphoviridae | Staphylococcus phage StauST398-4 |
| I2048_Contig_150 | 3.41690422 | 0.04962466 | Microviridae | Microviridae Fen418_41 |
| I2056_Contig_1639 | 3.40817269 | 0.00766521 | Siphoviridae | Bacillus phage PfEFR-4 |
| I2069_Contig_15 | 3.28850153 | 0.00375949 | Siphoviridae | Lactobacillus phage phiadh |
| I2056_Contig_1652 | 3.28468777 | 0.01488788 | Siphoviridae | Staphylococcus virus 108PVL |
| I2056_Contig_1600 | 3.2417663 | 0.00766521 | Siphoviridae | Staphylococcus prophage phiPV83 |
| I2056_Contig_12 | 3.19236585 | 0.01488788 | Siphoviridae | Bacillus phage PfEFR-4 |
| I2047_Contig_121 | 3.13522155 | 0.00766521 | Siphoviridae | Paenibacillus virus Vegas |
| I2056_Contig_1520 | 3.13136348 | 0.00766521 | Siphoviridae | Bacillus phage PfEFR-4 |
| I2060_Contig_128 | 3.08598936 | 0.01488788 | Myoviridae | Pseudomonas phage phi3 |
| I2072_Contig_1402 | 3.06659037 | 0.01488788 | Siphoviridae | Bacillus phage PfEFR-4 |
| I2069_Contig_1154 | 3.06127095 | 0.0276947 | Myoviridae | Lactobacillus prophage Lj771 |
| I2056_Contig_1624 | 3.05824572 | 0.00766521 | Siphoviridae | Staphylococcus phage StauST398-4 |
| I2060_Contig_13 | 3.05565568 | 0.01488788 | Siphoviridae | Bacillus phage PfEFR-4 |
| I2054_Contig_1320 | 2.99872686 | 0.0276947 | Siphoviridae | Paenibacillus virus Vegas |
| I2055_Contig_159 | 2.98478417 | 0.0276947 | Myoviridae | Bacillus virus G |
| I2053_Contig_113 | 2.98245336 | 0.0276947 | Unclassified | Unclassified |
| I2072_Contig_1315 | 2.97931291 | 0.04962466 | Myoviridae | Klebsiella virus K64-1 |
| I2054_Contig_1394 | 2.9562001 | 0.00766521 | Myoviridae | Brevibacillus virus Davies |
| I2072_Contig_1314 | 2.92484933 | 0.04962466 | Herelleviridae | Bacillus virus Shanette |
| I2055_Contig_130 | 2.91168766 | 0.04962466 | Siphoviridae | Planktothrix phage PaV-LD |
| I2060_Contig_1114 | 2.90130079 | 0.0276947 | Unclassified | Unclassified |
| I2054_Contig_1152 | 2.89957803 | 0.00766521 | Siphoviridae | Bacillus phage PfEFR-4 |
| I2055_Contig_17 | 2.88596456 | 0.0276947 | Herelleviridae | Lactobacillus virus Lb338-1 |
| I2069_Contig_1162 | 2.86877981 | 0.0276947 | Siphoviridae | Lactobacillus prophage Lj965 |
| I2056_Contig_1527 | 2.86073937 | 0.04962466 | Podoviridae | Sodalis phage phiSG1 |
| I2072_Contig_11756 | 2.8527256 | 0.0276947 | Myoviridae | Cronobacter phage vB_CsaM_GAP32 |
| I2054_Contig_178 | 2.85214976 | 0.04962466 | Siphoviridae | Staphylococcus phage StauST398-4 |
| I2054_Contig_1811 | 2.840441 | 0.04962466 | Siphoviridae | Staphylococcus virus 108PVL |
| I2056_Contig_1616 | 2.83112548 | 0.0276947 | Siphoviridae | Synechococcus phage S-CBS1 |
| I2056_Contig_185 | 2.80894665 | 0.01488788 | Siphoviridae | Staphylococcus phage SPbeta-like |
| I2072_Contig_1516 | 2.80330426 | 0.01488788 | Siphoviridae | Bacillus phage PfEFR-4 |
| I2048_Contig_1372 | 2.79279732 | 0.04962466 | Myoviridae | Prochlorococcus phage Syn33 |
| I2049_Contig_1691 | 2.7856956 | 0.04962466 | Siphoviridae | Lactobacillus prophage Lj928 |
| I2051_Contig_1850 | 2.7791778 | 0.0276947 | Podoviridae | Burkholderia virus Bcep22 |
| I2072_Contig_11597 | 2.77116786 | 0.0276947 | Myoviridae | Synechococcus phage S-PM2 |
| I2049_Contig_13 | 2.76742753 | 0.0276947 | Siphoviridae | Clostridium phage phiCTP1 |
| I2055_Contig_1975 | 2.7548439 | 0.04962466 | Siphoviridae | Planktothrix phage PaV-LD |
| I2054_Contig_1218 | 2.75421247 | 0.01488788 | Siphoviridae | Bacillus phage PfEFR-4 |
| I2065_Contig_183 | 2.74845045 | 0.04962466 | Herelleviridae | Lactobacillus virus Lb338-1 |
| I2048_Contig_1139 | 2.74414799 | 0.0276947 | Siphoviridae | Arthrobacter virus Kellezzio |
| I2065_Contig_1118 | 2.74342668 | 0.04962466 | Siphoviridae | Mycobacterium phage PegLeg |
| I2054_Contig_1532 | 2.74078253 | 0.04962466 | Siphoviridae | Clostridium phage c-st |
| I2060_Contig_129 | 2.72045995 | 0.04962466 | Siphoviridae | Staphylococcus phage StauST398-4 |
| I2072_Contig_1885 | 2.71990912 | 0.04962466 | Siphoviridae | Staphylococcus virus 108PVL |
| I2060_Contig_155 | 2.71206044 | 0.0276947 | Unclassified | Unclassified |
| I2072_Contig_1341 | 2.70701517 | 0.04962466 | Siphoviridae | Planktothrix phage PaV-LD |
| I2065_Contig_11176 | 2.703324 | 0.04962466 | Siphoviridae | Planktothrix phage PaV-LD |
| I2054_Contig_1227 | 2.69695854 | 0.04962466 | Herelleviridae | Enterococcus virus EFDG1 |
| I2072_Contig_1423 | 2.69437771 | 0.04962466 | Siphoviridae | Lactococcus phage 949 |
| I2072_Contig_11658 | 2.69169287 | 0.04962466 | Siphoviridae | Staphylococcus virus 108PVL |
| I2049_Contig_1107 | 2.66193949 | 0.04962466 | Herelleviridae | Bacillus virus Shanette |
| I2056_Contig_1654 | 2.64162813 | 0.04962466 | Siphoviridae | Staphylococcus phage StauST398-4 |
| I2072_Contig_1422 | 2.64099929 | 0.0276947 | Siphoviridae | Paenibacillus virus Vegas |
| I2055_Contig_1968 | 2.63827754 | 0.04962466 | Siphoviridae | Lactococcus phage Q54 |
| I2072_Contig_1486 | 2.62982163 | 0.0276947 | Siphoviridae | Bacillus phage vB_BtS_BMBtp3 |
| I2056_Contig_1659 | 2.62254076 | 0.0276947 | Siphoviridae | Paenibacillus phage Xenia |
| I2054_Contig_1210 | 2.60401944 | 0.0276947 | Myoviridae | Erwinia phage vB_EamM_Phobos |
| I2051_Contig_1922 | 2.59571666 | 0.0276947 | Siphoviridae | Clostridium phage phiCTP1 |
| I2048_Contig_136 | 2.59419569 | 0.04962466 | Myoviridae | Klebsiella virus K64-1 |
| I2056_Contig_1557 | 2.58819052 | 0.04962466 | Siphoviridae | Planktothrix phage PaV-LD |
| I2054_Contig_128 | 2.57208169 | 0.04962466 | Siphoviridae | Paenibacillus virus Lily |
| I2048_Contig_1181 | 2.56935226 | 0.04962466 | Myoviridae | Brevibacillus phage Jimmer1 |
| I2048_Contig_1650 | 2.54026109 | 0.04962466 | Siphoviridae | Bacillus virus SPbeta |
| I2051_Contig_1892 | 2.52856853 | 0.04962466 | Myoviridae | Klebsiella virus K64-1 |
| I2051_Contig_1142 | 2.52748699 | 0.04962466 | Siphoviridae | Planktothrix phage PaV-LD |
| I2060_Contig_138 | 2.52536916 | 0.04962466 | Siphoviridae | Bacillus phage PfEFR-4 |
| I2051_Contig_189 | 2.49987375 | 0.04962466 | Siphoviridae | Planktothrix phage PaV-LD |
| I2048_Contig_156 | 2.49168875 | 0.04962466 | Podoviridae | Burkholderia virus Bcep22 |
| I2054_Contig_112 | 2.49033768 | 0.0276947 | Myoviridae | Klebsiella virus K64-1 |
| I2054_Contig_1326 | 2.47332415 | 0.0276947 | Myoviridae | Bacillus virus G |
| I2053_Contig_184 | 2.46564554 | 0.04962466 | Herelleviridae | Lactobacillus virus Lb338-1 |
| I2060_Contig_1345 | 2.46551751 | 0.04962466 | Myoviridae | Bacillus virus G |
| I2048_Contig_1118 | 2.45926237 | 0.04962466 | Siphoviridae | Lactobacillus phage Ldl1 |
| I2048_Contig_169 | 2.44878167 | 0.04962466 | Siphoviridae | Bacillus phage PfEFR-4 |
| I2048_Contig_1694 | 2.43991777 | 0.04962466 | Myoviridae | Pseudomonas phage phi3 |
| I2054_Contig_1388 | 2.41404747 | 0.04962466 | Myoviridae | Bacillus virus G |
| I2048_Contig_1105 | 2.41327409 | 0.0276947 | Siphoviridae | Paenibacillus phage Xenia |
| I2072_Contig_11518 | 2.41021071 | 0.04962466 | Siphoviridae | Lactobacillus phage phiadh |
| I2053_Contig_1146 | 2.4043656 | 0.04962466 | Siphoviridae | Planktothrix phage PaV-LD |
| I2060_Contig_1419 | 2.40071279 | 0.04962466 | Myoviridae | Brevibacillus virus Davies |
| I2051_Contig_1915 | 2.38702307 | 0.04962466 | Siphoviridae | Planktothrix phage PaV-LD |
| I2053_Contig_194 | 2.38605929 | 0.04962466 | Siphoviridae | Staphylococcus phage StauST398-4 |
| I2051_Contig_1111 | 2.37367166 | 0.04962466 | Herelleviridae | Bacillus virus CP51 |
| I2072_Contig_1254 | 2.36837499 | 0.04962466 | Podoviridae | Cellulophaga phage phi14:2 |
| I2053_Contig_112 | 2.36794576 | 0.04962466 | Siphoviridae | Staphylococcus virus 42e |
| I2048_Contig_184 | 2.35407134 | 0.04962466 | Siphoviridae | Planktothrix phage PaV-LD |
| I2072_Contig_1206 | 2.34848148 | 0.04962466 | Siphoviridae | Bacillus virus SPbeta |
| I2054_Contig_122 | 2.34807031 | 0.04962466 | Myoviridae | Aeromonas virus 31 |
| I2072_Contig_132 | 2.34487258 | 0.04962466 | Siphoviridae | Mycobacterium phage Enkosi |
| I2053_Contig_1139 | 2.34410591 | 0.0276947 | Myoviridae | Aeromonas virus 31 |
| I2054_Contig_197 | 2.33714847 | 0.0276947 | Siphoviridae | Planktothrix phage PaV-LD |
| I2054_Contig_110 | 2.33666831 | 0.04962466 | Siphoviridae | Lactobacillus phage Ldl1 |
| I2072_Contig_1931 | 2.33449831 | 0.04962466 | Siphoviridae | Streptomyces virus Jay2Jay |
| I2053_Contig_1125 | 2.33035361 | 0.04962466 | Siphoviridae | Lactobacillus phage JCL1032 |
| I2051_Contig_1744 | 2.32471424 | 0.04962466 | Siphoviridae | Planktothrix phage PaV-LD |
| I2054_Contig_11219 | 2.32250503 | 0.04962466 | Myoviridae | Cronobacter phage vB_CsaM_GAP32 |
| I2054_Contig_160 | 2.32031481 | 0.04962466 | Myoviridae | Enterobacteria phage vB_KleM-RaK2 |
| I2072_Contig_1209 | 2.25449774 | 0.04962466 | Siphoviridae | Paenibacillus virus Vegas |
| I2054_Contig_1936 | 2.25194743 | 0.04962466 | Siphoviridae | Paenibacillus virus Diva |
| I2072_Contig_11513 | 2.22571338 | 0.04962466 | Siphoviridae | Bacillus phage vB_BanS-Tsamsa |
| I2072_Contig_1225 | 2.21148631 | 0.04962466 | Myoviridae | Bacillus phage 0305phi8-36 |
| I2048_Contig_195 | 2.18899247 | 0.04962466 | Myoviridae | Bacillus virus G |
| I2072_Contig_1933 | 2.16840642 | 0.04962466 | Myoviridae | Bacillus virus G |
